# Palliative Care Outcome Scale Assessment for Cancer Patients Eligible for Palliative Care: Perspectives on the Relationship between Patient-Reported Outcome and Objective Assessments

**DOI:** 10.3390/curroncol29100561

**Published:** 2022-09-28

**Authors:** Nobuhisa Nakajima

**Affiliations:** Division of Community Medicine and International Medicine, University of the Ryukyus Hospital, Okinawa 903-0215, Japan; nakajy@med.u-ryukyu.ac.jp

**Keywords:** patient-reported outcome, palliative care, integrated palliative care outcome scale (IPOS), support team assessment schedule (STAS)

## Abstract

(1) Background: The importance of patient-reported outcome (PRO), i.e., prioritizing patient voice, has increased in cancer treatment, as well as palliative and supportive settings. The Integrated Palliative Care Outcome Scale (IPOS), a hybrid evaluation consisting of “patient evaluation” (PRO) and “peer evaluation” by medical professionals, was developed as a successor version of the Support Team Assessment Schedule (STAS) in 2013 and has been utilized worldwide. The Japanese version of the IPOS (IPOS-J) was developed and released in 2019. The purpose of this study was to explore the applicability of the IPOS-J to clinical practice in the future. (2) Methods: We conducted the following two studies with terminally ill cancer patients: (i) Can an evaluation with the IPOS-J performed by medical professionals (peer evaluation) replace the STAS-J evaluation? (ii) Can the quality of palliative care improve by combining the IPOS-J patient evaluation with the peer evaluation? (3) Results: The overall intervention rate and urgent intervention rate for the STAS-J and IPOS-J was 34.4 vs. 34.1% (*p* = 0.91) and 10.4 vs. 9.9% (*p* = 0.78), respectively. The patients selected “intervention required” but the medical professionals selected “no intervention required” in 47 cases. The medical team performed appropriate intervention after re-assessment. As a result, more than 70% of the patients were “intervention-free” after 1 week of intervention. (4) Conclusions: The IPOS-J peer evaluation was as useful as the STAS-J evaluation. A hybrid type of evaluation, combining patient evaluation (PRO) and peer evaluation, may help us to understand patient needs and improve the quality of palliative care.

## 1. Introduction 

In the field of palliative care, various assessment tools are used in the clinical and research settings to evaluate the physical and psychiatric symptoms and quality of life of patients. Among them, the Support Team Assessment Schedule (STAS), a representative clinical audit tool based on peer evaluation, is used by medical professionals. It was developed by Higginson et al. in the UK in the 1980s [1,2] and is used worldwide [3,4]. 

The popularity of peer evaluation may be accounted for by several factors. First, palliative care patients tend to be in poor general health, making it difficult for them to participate in interviews or to use self-report-based tools. Second, the goal of palliative care is to alleviate the holistic suffering of patients and their families. Third, palliative care usually involves multidisciplinary teams; it is necessary to evaluate whether these team function well and place patients at the center of their activities. The Japanese version of the STAS (STAS-J) was published in 2004 [5] and is now widely used in clinical practice in Japan. Several previous studies have used this tool in a research context [6,7,8,9,10]. 

The importance of patient-reported outcome (PRO), i.e., prioritizing patient voice, has increased in cancer treatment, including in surgery, chemotherapy, and radiation therapy, as well as in palliative and supportive care [11]. PROs in the field of palliative care include the Edmonton Symptom Assessment System and the MD Anderson Symptom Inventory [12,13]; Japanese versions of both tools have been developed [14,15]. 

The Palliative Care Outcome Scale was developed in 1999 by Higginson et al. as a successor of the STAS [16]. Patient evaluation is an essential part of PRO assessments. The Integrated Palliative Care Outcome Scale (IPOS) was developed in 2013 [17] and is based on a combination of patient self-evaluation and medical professional peer evaluation. The tool has evolved since its creation [17] and is used worldwide in both the clinical and research settings [18,19]. 

A Japanese version of the IPOS (IPOS-J) was published in 2019 [20]. We have decided to propose the following questions in anticipation of a future transition from the STAS-J:

(1) Can an evaluation using the IPOS-J performed by medical professionals (peer evaluation) replace the STAS-J evaluation?
(2) Can the quality of palliative care improve by combining the IPOS-J patient evaluation, which is a PRO evaluation, with the peer evaluation?

The overall aim of this study was to explore the potential applicability of the IPOS-J (provisional version) in clinical practice. 

## 2. Methods 

We conducted two retrospective studies on consecutive terminally ill cancer patients admitted to the palliative care unit at Tohoku University Hospital between April 2014 and March 2016. First, we compared the STAS-J and IPOS-J (peer evaluation) scores. Second, we evaluated whether these differences affected the provided care and whether any changes in provided intervention affected the subsequent patient evaluation scores. 

The STAS-J assessment items and scoring criteria were as follows: (1) Pain, (2) Other symptoms, (3) Anxiety, (4) Family anxiety, (5) Insight, (6) Family insight, (7) Communication between patient and family, (8) Communication between professionals, and (9) Communication between patient/family and professionals. Each problem and need for improvement were scored on a 5-point (0–4) scale. High scores indicated several problems, and low scores indicated few problems. Table 1 presents an example evaluation for pain control. 

The IPOS-J assessment items and scoring criteria were as follows: (1) Pain, (2) Other symptoms, (3) Anxiety, (4) Family anxiety, (5) Depressive feelings, (6) Feeling at peace, (7) Sharing with family, friends, (8) Sufficient information by physicians, and (9) Practical (psychosocial) problems. The IPOS items were scored on a 5-point (0–4) scale. The specific scoring method is shown in Table 2. 

### 2.1. Preparation and Assessment Methods 

The author was involved in the development, revision, dissemination, and evaluation of the STAS-J [8,9,10]. Regarding the IPOS, the author had the opportunity to learn this tool at King’s College London, where the tool was created in 2013, and was involved in the development process of the IPOS-J. After the IPOS-J was released, ward staff who were familiar with the STAS-J evaluation were offered the opportunity to receive adequate training on the IPOS-J, helping in establishing a reliable evaluation system. 

The author, together with these staff members, used the STAS-J and IPOS-J for initial peer evaluation of patients within a few days of admission and continued these evaluations 1–2 times per week thereafter. The staff assisted in the IPOS patient evaluation, as required. As in the case of peer evaluation by medical professionals, the initial evaluation was performed within a few days of admission and was continued 1–2 times a week thereafter. 

### 2.2. Comparison of the STAS-J and IPOS-J (Peer Evaluation) Scores 

Similar items from the STAS-J and IPOS-J tools were considered. Items (1), (2), (3), (4), (5), (7), and (9) of the STAS-J were judged to correspond to items (1), (2), (3), (4), (8), and (9) of the IPOS-J. None of the IPOS-J items corresponded to “family insight” and “communication between professionals” in the STAS-J. Meanwhile, the IPOS-J items “anxiety,” “depressive feeling,” and “calmness” corresponded to “insight” on the STAS-J. 

Using the STAS-J and IPOS-J scores, we classified the results into three categories: no intervention required (0–1), intervention required (2), and immediate intervention required (3–4), which reflected the need and urgency of intervention required. We then compared the intervention required and immediate intervention required rates between the two tools. 

### 2.3. Changes in the Evaluation Scores after Implementation of the Intervention Based on Differences in Scores 

Based on the two IPOS-J evaluation (patient evaluation and peer evaluation), we classified the results into three categories: (1): no intervention required (0–1), intervention required (2), and immediate intervention required (3–4). We compared the intervention required and immediate intervention required rates between these two evaluation methods. Next, when the patient evaluation score was higher than the peer evaluation score, i.e., when the intervention need expressed by the patient was larger than that noted by a medical professional, we reviewed the content of the intervention performed after determining within the medical team on whether an intervention was necessary and whether the intervention content should be changed. We then investigated the content of the intervention that were performed. Finally, we compared the changes in patient evaluation scores before and 1 week after the intervention. 

### 2.4. Statistical Analysis

The need for intervention rates were compared using the chi-square test. *p*-values < 0.05 were considered statistically significant.

## 3. Results

Since the IPOS-J lacked items corresponding to the STAS-J items “family anxiety” and “communication between medical professionals,” these items were excluded from the evaluation. The scores for the seven remaining items were compared between the STAS-J and IPOS-J (n = 102 patients). In the case of “insight,” “communication between patient and family,” and “communication between patient/family and medical professionals,” 8, 11, and 12 patients could not be evaluated due to delirium, impaired consciousness or sedation, respectively; 94, 86, and 90 patients were evaluated, respectively. 

Overall, “no intervention required (0–1),” “intervention required (2),” and “immediate intervention required (3–4)” findings were consistent between the two evaluation methods in 596/612 (97.3%) cases. The consistency rates for findings of pain, other symptoms, anxiety, family anxiety, communication between patient and family, and communication between patient/family and medical professionals were 96.0% (98/102), 97.0% (99/102), 94.1% (96/102), 100% (102/102), 100% (102/102), and 97.0% (99/102), respectively. 

The number of patients who needed an intervention (2) according to the STAS-J but did not need any intervention (0–1) according to the IPOS-J was 3 (pain: 1 case, other symptoms: 2 cases). Three patients required “no intervention (0–1)” according to the STAS-J, but required an intervention (2) according to the IPOS-J for anxiety (2 cases) and communication between patient/family and medical professionals (1 case). Three patients required an immediate intervention for pain (3–4) according to the STAS-J and needed an intervention (2) on the IPOS-J. One patient required an immediate intervention for pain and four patients required immediate interventions for anxiety (3–4) according to the IPOS-J; in contrast, the intervention in these cases was not considered urgent according to the STAS-J. 

The overall need for intervention rates for the STAS-J and IPOS-J were 34.4 vs. 34.1% (*p* = 0.91), respectively. The corresponding rates for pain, other symptoms, anxiety, family anxiety, insight, communication (patient and family), and communication were 33.3 vs. 34.3% (*p* = 0.88), 47.0 vs. 46.0% (*p* = 0.89), 43.1 vs. 41.6% (*p* = 0.78), 38.1 vs. 38.1% (*p* > 0.99), 2.8 vs. 12.8% (*p* > 0.99), 26.7 vs. 26.7%, % (*p* > 0.99), and 37.8 vs. 37.8% (*p* > 0.99), respectively.

The overall STAS-J and IPOS-J rates of urgent intervention need were 10.4 vs. 9.9% (*p* = 0.78), respectively. The corresponding values for pain, other symptoms, anxiety, family anxiety, insight, communication (patient and family), and communication (patient/family and medical professionals) were 23.5 vs. 21.6% (*p* = 0.74), 15.7 vs. 16.7% (*p* = 0.85), 12.7 vs. 16.7% (*p* = 0.43), 6.4 vs. 6.4% (*p* > 0.99), 0 vs. 0% (*p* > 0.99), 5.6 vs. 5.6% (*p* > 0.99), and 0% vs. 0% (*p* > 0.99), respectively. (Table 3)

The “no intervention required (0–1),” “intervention required (2),” and “immediate intervention required (3–4)” scores were consistent between patient evaluation and peer evaluation in 83.6% (564/674) of cases. The corresponding score agreements for pain, other symptoms, anxiety, depression, feeling at peace, sharing, sufficient information, and practical problems were 87.5% (77/87), 83.9% (73/87), 82.8% (72/87), 85.1% (74/87), 83.9% (73/87), 86.1% (68/79), 79.0% (64/81), and 79.7% (63/79), respectively. 

In contrast, interventions were deemed as required by patients but not by medical professionals in 47 cases (3, 4, 7, 5, 3, 4, 13 [3 for immediate intervention], and 8 [3 for immediate intervention] cases for pain, other symptoms, anxiety, depressive feeling, feeling at peace, well sharing, sufficient information, and practical problems, respectively).

Finally, interventions were deemed as not required by patients and as required by medical professionals in 25 cases (2, 2, 3, 3, 5, 3, 2, and 5 cases for pain, other symptoms, anxiety, depressive feeling, feeling at peace, well sharing, sufficient information, and practical problems, respectively). The number of patients with “immediate intervention needed (3–4)” according to the patient evaluation but “intervention needed (2)” by peer evaluation was 23 (3, 4, 3, 2, 4, 2, 2, and 3 cases for pain, other symptoms, anxiety, depressive feeling, feeling at peace, well sharing, sufficient information, and practical problems, respectively). The number of patients who rated as “intervention needed (2)” for themselves and as “urgent intervention needed (3–4)” by the medical professionals was 15 (2, 4, 2, 3, 2, and 2 cases for pain, other symptoms, anxiety, depressive feeling, feeling at peace, and well sharing, respectively). 

When the patient evaluation score was higher than the peer evaluation score, i.e., the patient-judged need for intervention was larger than the professional-judged need, the medical team evaluated the need for and content of the intervention, which was implemented, as required. 

The number of cases in which the patient assessment score 1 week after the intervention improved from “intervention required (2)” or “immediate intervention required (3- 4)” to “no intervention required (0–1)” was 2 of 3 for pain with opioid titration, aggressive use of rescue doses, and addition of analgesic adjuvant. For other symptoms, 3 of 4 patients required no intervention after treatment with steroids, opioids, or anti-emesis, among others. Anxiety, depressive feeling, and feeling at peace required no intervention in 5 of 7, 3 of 5, and 2 of 3 patients, respectively, after medication and psychosocial support. “Well sharing with family and friends,” “sufficient information by physicians,” and “practical problem” items required “no intervention (0–1)” after informed consent and social support provisions were introduced in 3 of 4 cases, 10 of 13 cases, and 5 of 8 cases, respectively. In addition, 3 of 3 and 2 of 3 patients requiring an “urgent intervention (3–4)” for sufficient information by physicians and practical problems, respectively, required “no intervention (0–1)” after treatment. (Table 4)

## 4. Discussions 

This study aimed to assess whether the peer evaluation of the IPOS-J by medical professionals could replace the evaluation by the STAS-J. This study assessed 102 patients with terminal cancer admitted to a palliative care unit. Three patients with “intervention required (2)” according to the STAS-J but “intervention not required (0–1)” according to the IPOS-J score were at risk of missing the opportunity for an intervention. The overall agreement rate of “no intervention/need intervention/urgent intervention” was 97.3%, and the rate of intervention and urgent intervention required were comparable between the scores. It is necessary to take into consideration that there are no items in the IPOS-J that correspond to the two items of the STAS-J, “family anxiety” and “communication between medical professionals”. On the other hand, the IPOS-J assesses three dimensions of psychiatric symptoms (anxiety, depressive feeling, and calmness); in contrast, the STAS-J assesses only one (anxiety) such dimension. The IPOS-J examines psychiatric symptoms from several perspectives, and it may be more clinically useful than the STAS-J. Considering these factors, the IPOS-J was considered as clinically useful as the STAS-J when used as a substitute of the STAS-J.

The second aim of this study was to evaluate whether the quality of palliative care provided improved by combining patient and peer evaluation. The IPOS-J peer evaluation and STAS-J evaluation are of similar quality. The agreement on intervention need between the scores was 83.6%; the lowest agreement rate (79.0%) was noted for “sufficient information by physicians.” This finding suggests that physicians may believe that they provide sufficient information to patients, while patients require more information. The second lowest agreement rate was for practical problems at 79.3%. This finding suggests that medical professionals should take more time to evaluate the patient social context and to confirm whether the offered intervention is satisfactory. 

Among 47 cases in which peer and patient evaluation revealed “no need” and a “need” for an intervention, respectively, the following interventions were implemented after reevaluation, as suitable. Pain and dyspnea were treated with medication, while psychiatric symptoms were treated with medication and psychosocial intervention. For “sharing with family and friends,” “sufficient information by physicians,” and “practical problems,” informed consent and social support frameworks were provided. Consequently, more than 70% of the patients required “no intervention” after a week of treatment. This included 5 of 6 cases, in which patients required an urgent intervention for “sufficient information by physicians” and “practical problems.” 

A combination of peer and patient evaluations may help understand patient needs and improve patient care, alleviating suffering at the end of life. We believe that these observations need to be verified in the future through prospective intervention studies. 

The concepts of “early palliative care” [10,21] and “integration of oncology and palliative care” [22] have recently become popular in the field of cancer care. In this context, we expect that the IPOS-J, a hybrid assessment tool, will enable us to provide high-quality palliative care at an earlier stage of cancer treatment and to support the transition from cancer treatment to palliative care at an appropriate time with respect for the patient’s wishes and with enhanced social support.

This study has the following limitation. The STAS-J and IPOS-J scores were evaluated by the same evaluator. This may have introduced intra-rater bias, which must be considered when interpreting the results of this study. 

## 5. Conclusions

The IPOS-J peer evaluation was as useful as the STAS-J evaluation. Therefore, it was deemed appropriate to replace the STAS-J with the IPOS-J. Furthermore, by adding patient evaluation (PRO) to peer evaluation and emphasizing “listening to the patient’s voice,” it will be possible to further understand the problems that patients face and to more accurately assess the extent of these problems. Appropriate intervention based on this will enable the provision of higher quality palliative care. It is important to verify these observations through prospective studies and to promote the widespread use of this tool.

## Figures and Tables

**Table 1:** Pain control: Effect of his/her pain on the patient.

0 = None.
1 = Occasional or grumbling single pain. Patient is not bothered to be rid of symptom.
2 = Moderate distress, occasional bad days, pain limits some activity possible within extent of disease.
3 = Severe pain present often. Activities and concentration markedly affected by pain.
4 = Severe and continuous overwhelming pain. Unable to think of other matters.

**Table 2:** The IPOS-J assessment items and scoring criteria.

(1) Pain, (2) Other symptoms;
0—Not at all, 1—Slightly, 2—Moderately, 3—Severely, 4—Overwhelmingly,
(3) Anxiety, (4) Family anxiety, (5) Depressive feeling;
0—Not at all, 1—Occasionally, 2—Sometimes, 3—Most of the time, 4—Always,
(6) Feeling at peace, (7) Well sharing with family, friends, (8) Sufficient information by physicians (clinicians);
0—Always, 1—Most of the time, 2—Sometimes, 3—Occasionally, 4—Not at all,
(9) Practical problems (psycho, social);
0—Problems addressed/no problem, 1—Problems mostly addressed, 2—Problems partly addressed, 3—Problems hardly addressed, 4—Problems not addressed

**Table 3:** Comparison of the STAS-J and IPOS-J medical assessment score.

Item of STAS-J	Item of IPOS		IPOS Score		
		STAS-J Score	0–1	2	3–4
Pain	Pain	0–1	44	0	0
		2	1	32	1
		3–4	0	3	21
Other symptoms	Other symptoms	0–1	38	0	0
		2	2	46	0
		3–4	0	1	5
Anxiety	Anxiety	0–1	43	2	0
	Depressive feeling	2	0	40	4
	Calmness	3–4	0	0	13
Family anxiety	Family anxiety	0–1	58	0	0
		2	0	37	0
		3–4	0	0	6
Insight	Sufficient information	0–1	82	0	0
	by physicians (clinicians)	2	0	12	0
		3–4	0	0	0
Communication-1 *	Well sharing	0–1	62	0	0
	with family and friends	2	0	24	0
		3–4	0	0	5
Communication-2 **	Practical problems (psycho, social)	0–1	53	1	0
		2	2	34	0
		3–4	0	0	0

Remarks: * Communication-1 means Communication between patient and family, ** Communication-2 means Communication between patient/family and professionals.

**Table 4:** Comparison of the peer and patient IPOS-J assessment score.

Item of STAS-J	Evaluation		Peer evaluation (Medical Professionals)			Remarks
			0–1	2	3–4	Contents of Intervention
Pain	Patient	0–1	35	2	0	opioid titration
		2	3	26	2	rescue dose
		3–4	0	3	16	analgesic adju
Other symptoms		0–1	27	2	0	dyspnea, general fatigue
		2	4	35	4	abd. Distention
		3–4	0	4	11	medication, (steroid, opioid)
Anxiety		0–1	31	3	0	medication
		2	7	29	2	psychosocial support
		3–4	0	3	12	
Depressive feeling		0–1	37	3	0	medication
		2	5	27	3	psychosocial support
		3–4	0	2	10	
Feeling at peace		0–1	41	5	0	psychosocial support
		2	3	22	2	
		3–4	0	4	10	
Well sharing with family, friends		0–1	52	3	0	informed consent
		2	4	13	2	social support
		3–4	0	2	3	
Sufficient information by physicians		0–1	56	2	0	informed consent
		2	10	8	0	
		3–4	3	2	0	
Practical problems (including psycho, social)		0–1	47	5	0	social support
		2	5	16	0	
		3–4	3	3	0	

## Data Availability

The data presented in this study are available on request from the corresponding author.
